# CD3^+^CD4^+^gp130^+^ T Cells Are Associated With Worse Disease Activity in Systemic Lupus Erythematosus Patients

**DOI:** 10.3389/fimmu.2021.675250

**Published:** 2021-06-04

**Authors:** Nur Diyana Mohd Shukri, Aziz Farah Izati, Wan Syamimee Wan Ghazali, Che Maraina Che Hussin, Kah Keng Wong

**Affiliations:** ^1^ Department of Immunology, School of Medical Sciences, Universiti Sains Malaysia, Kubang Kerian, Malaysia; ^2^ Hospital Universiti Sains Malaysia, Kubang Kerian, Malaysia; ^3^ Department of Internal Medicine, School of Medical Sciences, Universiti Sains Malaysia, Kubang Kerian, Malaysia

**Keywords:** systemic lupus erythematosus, gp130, *IL6ST*, IL-35, IL-12Rβ2, SLEDAI-2K

## Abstract

The receptors for IL-35, IL-12Rβ2 and gp130, have been implicated in the inflammatory pathophysiology of autoimmune diseases. In this study, we set out to investigate the serum IL-35 levels and the surface levels of IL-12Rβ2 and gp130 in CD3^+^CD4^+^, CD3^+^CD4^─^ and CD3^─^CD4^─^ lymphocyte subpopulations in systemic lupus erythematosus (SLE) patients (n=50) versus healthy controls (n=50). The potential T cell subsets associated with gp130 transcript (*i.e. IL6ST*) expression in CD4^+^ T cells of SLE patients was also examined in publicly-available gene expression profiling (GEP) datasets. Here, we report that serum IL-35 levels were significantly higher in SLE patients than healthy controls (*p*=0.038) but it was not associated with SLEDAI-2K scores. The proportions of IL-12Rβ2^+^ and gp130^+^ cells in SLE patients did not differ significantly with those of healthy controls in all lymphocyte subpopulations investigated. Essentially, higher SLEDAI-2K scores were positively correlated with increased proportion of gp130^+^ cells, but not IL-12Rβ2^+^ cells, on CD3^+^CD4^+^ T cells (r=0.425, *p*=0.002, *q*=0.016). Gene Set Enrichment Analysis (GSEA) of a GEP dataset of CD4^+^ T cells isolated from SLE patients (n=8; GSE4588) showed that *IL6ST* expression was positively associated with genes upregulated in CD4^+^ T cells vs myeloid or B cells (*q*<0.001). In an independent GEP dataset of CD4^+^ T cells isolated from SLE patients (n=9; GSE1057), *IL6ST* expression was induced upon anti-CD3 stimulation, and that Treg, T_CM_ and CCR7^+^ T cells gene sets were significantly enriched (*q*<0.05) by genes highly correlated with *IL6ST* expression (n=92 genes; r>0.75 with *IL6ST* expression) upon anti-CD3 stimulation in these SLE patients. In conclusion, gp130 signaling in CD3^+^CD4^+^ T cell subsets may contribute to increased disease activity in SLE patients, and it represents a promising therapeutic target for inhibition in the disease.

## Introduction

Systemic lupus erythematosus (SLE) is a chronic systemic autoimmune disease characterized by loss of immune tolerance, production of autoantibodies, imbalanced cytokine levels and their downstream signaling pathways (1, 2). The interleukin-12 (IL-12) family of heterodimeric cytokines consists of IL-12, IL-23, IL-27 and IL-35 (3). Each member of the IL-12 family has been implicated in the development and severity of SLE in which the IL-12 and IL-23/Th17 axis are recognized therapeutic targets in the disease (4–6), and IL-27 is involved in the pathogenesis of SLE (7, 8).

IL-35 is a novel cytokine in the IL-12 family produced by regulatory T cells (Tregs), regulatory B cells (Bregs), activated dendritic cells and monocytes (9–12). It is similar with other IL-12 family of cytokines which function as heterodimers where each cytokine comprises of heterodimeric subunits (an α-subunit and a β-subunit). IL-35 exerts anti-inflammatory actions by inhibiting effector T cells (13) and restricts tissue injury by inducing the expansion of Treg and Breg cells (10, 11, 14). However, IL-35 also exerts pro-inflammatory effects by activating the production of IL-12 and IFN-γ (15), and its expression is upregulated in autoimmune diseases such as rheumatoid arthritis (RA) where it augments the release of pro-inflammatory factors such as IL-1β and IL-6 (16). In SLE, multiple studies have reported that serum IL-35 levels are increased in established SLE patients on treatment (17, 18).

The heterodimeric cytokine comprises of two subunits, the Epstein-Barr virus-induced gene protein 3 (EBI3) and p35 subunit of IL-12 (14, 19). The EBI3 subunit is shared with IL-27, and the p35 subunit is shared with IL-12 (20). The IL-35 receptors consist of IL-12 receptor subunit β2 (IL-12Rβ2) homodimers (IL-12Rβ2/IL-12Rβ2), glycoprotein 130 (gp130) homodimers (gp130/gp130) or IL-12Rβ2/gp130 heterodimers (15). IL-35 signaling is mediated either through the heterodimer of receptor chains (IL-12Rβ2/gp130) or the homodimer of each chain, where binding of the IL-35 to cognate receptors activates the receptor-associated JAK leading to activation of the JAK/STAT signaling cascade (9, 21).

The gp130 cytokine receptor family is utilized by numerous structurally related cytokines of the IL-12 and IL-6 (*e.g.* IL-6, IL-11, IL-27) family where at least one gp130 subunit binds to the cytokine to trigger the JAK/STAT pathway (22). gp130 is a crucial factor for hematopoiesis, activation of immune responses and maintaining a long-lasting antiviral immunity by CD4^+^ T cells (23–25). The receptor plays key roles in inflammation whereby stimulation of IL-6R/gp130 receptor complex by IL-6 on naïve CD4^+^ T cells triggers STAT3 activation for Th17 differentiation and effector function (26). In autoimmune diseases, hyperactive gp130/STAT signaling leads to augmented inflammatory arthritis through increased infiltrating T cells in the joints (27), and blockade of gp130 activities by therapeutic antibodies has been proposed as a potential therapy in RA (28). In SLE, it has recently been shown that gp130 expression is induced in a subset of Treg cells that have lost their suppressive function, and activation of gp130 is required to derail the suppressive capacity of human Treg cells (29). Nevertheless, it remains unclear if the IL-35/IL-12Rβ2/gp130 axis is associated with SLE in lymphocyte subpopulations.

In this study, we set out to determine the levels of serum IL-35 and surface levels of IL-12Rβ2 and gp130 in lymphocyte subpopulations *i.e.* CD3^+^CD4^+^, CD3^+^CD4^─^ and CD3^─^CD4^─^ cells of SLE patients compared with healthy controls. The associations of IL-35, IL-12Rβ2 and gp130 with SLE Disease Activity Index-2K (SLEDAI-2K score) were investigated. The potential CD4^+^ T cell subsets associated with *IL6ST* (interleukin 6 cytokine family signal transducer) expression levels in SLE patients were also examined *via* bioinformatics analysis.

## Materials and Methods

### Study Population

This study was a case–control study conducted from November 2018 until May 2019 at Rheumatology Clinic, Hospital Universiti Sains Malaysia (HUSM) and the research laboratory of Department of Immunology, Universiti Sains Malaysia (USM). Fifty patients who were diagnosed with SLE, according to the 1997 revised criteria of the American College of Rheumatology (ACR) or the 2012 Systemic Lupus International Collaborating Clinics (SLICC) criteria (30–32), were enrolled in this study. The disease activity of each patient was assessed using the SLEDAI-2K scores during follow-up by the attending physician. Healthy controls (n=50) were recruited from voluntary hospital staffs and students. The median age of the SLE cohort was 34.0 years and the control group was 26.5 years. This study is part of our interleukin receptors profiling project where the same subjects were recruited for PBMCs isolation before downstream experiments were conducted separately to investigate distinct biomarkers. As such, the clinico-demographical characteristics of the subjects are currently under review. Nonetheless, for clarity, the clinico-demographical characteristics of the subjects are presented in **Supplementary Table 1** whereby different parameters (*e.g.* treatment combinations) of the subjects’ characteristics were described. The patients’ SLEDAI-2K index values, proportion of anti-dsDNA positivity and dosage of concomitant steroids are also presented in **Supplementary Table 1**. In particular, of the 36 patients in our series of 50 SLE patients (73.4%) who received prednisolone, majority of the patients (n=30, 83.3%) received 5-10 mg of prednisolone once daily while the remaining six patients (16.7%) received 15-30 mg of prednisolone once daily. A total of 21 SLE patients had medical records describing the types of disease manifestations, if present, whereby five of them presented with lupus nephritis followed by four with skin manifestations, three with cardiac involvement, and two with neurologic manifestations.

### Ethics Statement

This study was approved by the Human Research Ethics Committee of USM (USM/JEPeM/17120680). The clinical and demographic data of the subjects such as age, gender and medications were obtained from interviews and medical records. All procedures performed in this study involving blood samples were according to the 1964 Declaration of Helsinki and its later updates and with the ethical standards of our institution. Written informed consent was attained from each participant after detailed explanations of the study. The written informed consent permitted the researchers to carry out the recruitment, drawing participant’s blood for experiments and publish the results. All samples were labeled anonymously and all data were recorded, stored and analyzed anonymously where none of the participant’s private information such as name was disclosed. All experimental procedures were carried out in accordance with the institutional guidelines and regulations.

### Samples Preparation and Peripheral Blood Mononuclear Cells (PMBCs) Isolation

Ten ml of whole blood was withdrawn from the peripheral vein of each recruited subject. Four ml of blood was placed in ethylenediaminetetraacetic acid (EDTA) tube for PBMCs isolation for flow cytometry analysis. The remaining 6 ml was placed in a clot activator tube, immediately centrifuged to obtain the serum by separating serum from fibrinogen (components of plasma) and cells, and stored at -80°C until further use. Each tube was labeled with the subject or specimen number, date and the intended test.

In the separate 4 ml of peripheral blood collected in the EDTA tube for flow cytometry analysis, it was kept in an upright position for at least 30 minutes at room temperature. The PBMCs were subsequently isolated by centrifugation against a density gradient using Histopaque-1077 (Sigma-Aldrich, USA). Briefly, the blood was transferred into a new falcon tube containing 4 ml of histopaque by carefully layering the blood onto the histopaque. The mixture of whole blood and histopaque was centrifuged for 30 minutes at 400 xg at room temperature (Eppendorf 5810, USA). After centrifugation, the thick white buffy coat layer was transferred using a Pasteur pipette into a new falcon tube. The cells were washed by adding 10 ml of isotonic phosphate-buffered saline (PBS; Oxoid, UK) solution and mixed gently by drawing in and out of the pipette. Next, the mixture was centrifuged at 250 xg for 10 minutes and the supernatant layer was aspirated and discarded. The cell pellet was suspended with 5 ml of isotonic PBS and mixed by gently drawing in and out with a Pasteur pipette. The mixture was centrifuged again at 250 xg for 10 minutes. After centrifugation, the supernatant was discarded and the cell pellet was resuspended in 500 µl PBS and was ready for cell counting. The PBMCs were adjusted to a final concentration of 2 x 10⁶ cells/ml by adding a calculated amount of cold fetal bovine serum (FBS) (Beckton Dickinson, USA). The cells were subsequently stained on the same day of the isolation for flow cytometry analysis.

### Flow Cytometry Analysis of Surface Receptors

For staining of surface receptors, 50 µl aliquots of the PBMCs (10⁶ cells) were dispensed into new microcentrifuge tubes where the cells were incubated with fluorochrome-labeled monoclonal antibodies (mAbs) and isotype-matched control antibodies (anti-human IgG). The following fluorochrome-labeled mouse mAbs (IgG1) were used at their recommended concentration (µl/10^6^ cells) according to manufacturer’s protocols: APC anti-IL-12Rβ2 (clone 305719; R&D Systems, USA; 10 µL/10^6^ cells), PE anti-gp130 (clone 28126; R&D Systems; 10 µL/10^6^ cells), APC-H7 anti-CD3 (clone SK7; Beckton Dickinson; 5 µl/10^6^ cells), and PE-Cy™7 anti-CD4 (clone SK3; Beckton Dickinson; 5 µl/10^6^ cells). Each tube was incubated with the antibodies for 30 minutes on ice and protected from light. Then, the cell suspensions were washed twice with cold FBS and centrifuged for 5 minutes at 1,000 rpm. The cell pellets were resuspended in 500 µl FBS and transferred into FACS tube prior to analysis. The stained samples were analyzed using the flow cytometer BD FACSCanto™ II (Beckton Dickinson) where a minimum of 10,000 events/tube was measured and the results were acquired using FACSDiva™ software. The FlowJo (version 10) software (TreeStar, USA) was used for downstream data analysis.

### Gating Strategy

**Figure 1:**
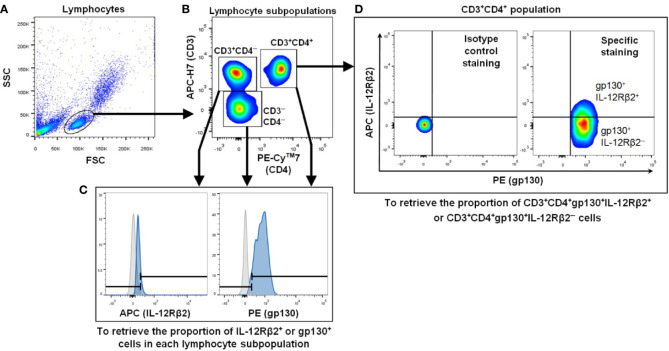
Representative flow cytometry gating strategy to measure IL-12Rβ2^+^ and gp130^+^ cells in lymphocyte subpopulations. **(A)** FSC and SSC were used to define the lymphocyte population. **(B)** Three lymphocyte subpopulations were gated *i.e.* CD3^─^CD4^─^, CD3^+^CD4^─^ and CD3^+^CD4^+^ populations. **(C)** Each lymphocyte subpopulation was examined for the proportion of IL-12Rβ2^+^ or gp130^+^ cells. The gray and blue histograms represent cells stained with isotype-matched control and IL-12Rβ2 or anti-gp130 mAbs, respectively. Both histograms guided the ranged gates to define IL-12Rβ2^+^ or gp130^+^ population *i.e.* the right gate for each protein. **(D)** Isotype control staining (left panel) and specific staining (gp130 and IL-12Rβ2 staining) (right panel) gated with the same cut-off for both PE (gp130) and APC (IL-12Rβ2) signals. Within CD3^+^CD4^+^gp130^+^ population, the proportion of IL-12Rβ2^+^ or IL-12Rβ2^─^ cells was determined (right panel).

For all fluorochromes studied (APC, PE, APC-H7 and PE-Cy7), the peak of the histogram for isotype controls were fixed at 1 (*i.e.* 10^0^). In order to differentiate the positive cells population from the negative population, the histograms of isotype control-stained samples were overlapped with the single color fluorochrome-stained samples. The lymphocytes were gated using forward scatter (FSC) and side scatter (SSC) followed by further gating on the lymphocytes to yield three subpopulations *i.e.* CD3^─^CD4^─^, CD3^+^CD4^─^ and CD3^+^CD4^+^, and to examine the proportion of these cells expressing surface IL-12Rβ2 or gp130 (**Figure 1**). The gate for gp130 (PE) or IL-12Rβ2 (APC) fluorescent signal was set at 300, the cut-off that distinguished each sample’s fluorescent intensity from that of the isotype control in the histogram. The levels of IL-12Rβ2 or gp130 in the CD3^─^CD4^+^ population could not be determined due to CD3^─^CD4^+^ population accounted for <1% of total lymphocytes.

### ELISA Measurement of Serum IL-35 Levels

Serum IL-35 levels were measured using Human IL-35 ELISA Kit (Cusabio, China) according to manufacturer’s instructions. This assay utilizes the quantitative sandwich enzyme immunoassay technique, and the microplate has been precoated with antibody specific for human IL-35. A total of 100 µl of standards and samples (in duplicate) were pipetted into the microplate wells and incubated for 2 hours at 37°C, and IL-35 present in the serum was bound by the immobilized antibody. After discarding the unbound substances by washing with wash buffer, 100 µl biotin-conjugated antibody specific for IL-35 was added to the wells and incubated for 1 hour at 37°C. After washing, 100 µl of avidin-conjugated horseradish peroxidase was added to the wells and incubated for 1 hour at 37°C. After the second wash to remove any unbound avidin-enzyme reagent, 90 µl of TMB substrate solution was added to the wells which changed the color of the solution in proportion to the amount of IL-35 bound in the initial step. Lastly, a stop solution was added to end the color development after 30 minutes at 37°C, protected from light, and the intensity of the color formed was immediately measured by an ELISA plate reader (Tecan, Switzerland) at 450 nm wavelength.

### Gene Expression Profiling (GEP) Datasets and Enrichment Analysis

Two independent microarray GEP datasets of CD4^+^ T cells isolated from SLE patients or healthy controls were obtained from the Gene Expression Omnibus database (http://www.ncbi.nlm.nih.gov/geo/) as follows: (i) GSE4588 (n=8 SLE patients) of CD4^+^ T cells sorted out by flow cytometry from PBMCs after staining with FITC-labeled anti-CD4 antibody; (ii) GSE1057 (n=9 for each SLE patients or healthy controls group) of CD4^+^ T cells isolated by anti-CD4 antibody-coupled magnetic beads. The CD4^+^ T cells were stimulated with anti-CD3 mAb (for CD4^+^ T cells derived from SLE and control subjects) or anti-CD3 mAb combined with anti-CD28 mAb (for CD4^+^ T cells derived from control subjects only) for 1h, 3h, 6h, 12h or 20h before anergy induction by resting the cells (*i.e.* without any stimulation) for 3 days before repeating the stimulation for 3h or 6h. For detailed protocols of the stimulation, readers are directed to the original publication by Xu et al. (33). Gene Set Enrichment Analysis (GSEA) (34) according to two independent *IL6ST* probe’s expression values (204863_s_at and 212195_at) and the C7 immunologic signature gene sets of Molecular Signatures Database (MSigDB) collections (35) was carried out for each of the *IL6ST* probe independently by adopting Pearson correlation as the gene-ranking metric and permutated with the gene sets permutation function (36–38). Functional enrichment analysis of genes associated with *IL6ST* expression (Pearson r > 0.75 or < -0.75 with *IL6ST*) in CD4^+^ T cells of SLE patients or controls (n=9 in each group; GSE1057) were conducted with the ToppGene Suite (39, 40). For both GSEA and ToppGene functional enrichment analysis, SLE gene sets with *p*-value following the Benjamini-Hochberg correction (*i.e. q*-value) below 0.05 were considered to be significant.

### Statistical Analysis

Unpaired t-test or Mann-Whitney U test was used for comparison between groups of normally or not normally distributed data according to the Shapiro-Wilk Test, respectively. Spearman correlation coefficient was used for correlation analysis between the proportion of lymphocyte subpopulations expressing IL-12Rβ2 or gp130 with SLEDAI-2K scores. A total of eight associations with SLEDAI-2K scores were tested by Spearman correlation coefficient where two of the associations yielded significance. In order to reduce type I error (*i.e.* false positive), we performed correction for multiple comparisons according to the Benjamini-Hochberg (BH) procedure, and BH-corrected *p*-value (*i.e. q*-value) below 0.05 was considered as significant. For comparisons of more than two groups, Dunn’s multiple comparisons *post hoc* test was conducted following Kruskal-Wallis test. All statistical analysis was performed using GraphPad Prism (v9; GraphPad Software Inc., USA), and two-tailed *p*<0.05 was considered to be statistically significant.

## Results

### Association of Serum IL-35 With SLE and SLEDAI-2K Scores

**Figure 2:**
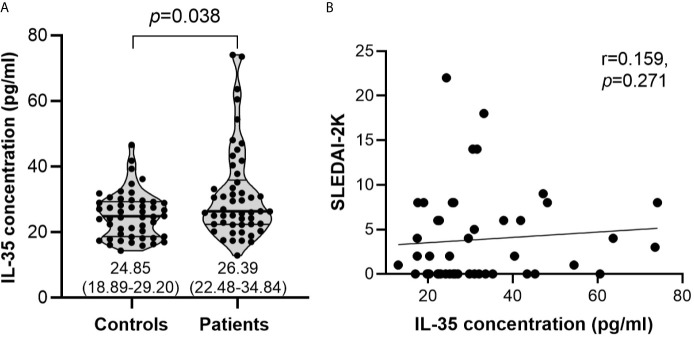
Serum IL-35 levels and their association with SLEDAI-2K scores. **(A)** Serum IL-35 levels (pg/ml) in healthy controls (n=50) vs SLE patients (n=50). The inset numbers below each violin plot represent median (IQR). **(B)** Association of serum IL-35 levels with SLEDAI-2K scores in SLE patients (n=50).

Serum IL-35 was elevated in SLE patients (median: 26.39 pg/ml; interquartile range, IQR: 22.48-34.84 pg/ml) compared with healthy controls (median: 24.85 pg/ml; IQR: 18.89-29.20 pg/ml) although with borderline significance (*p*=0.038) (**Figure 2A**). In terms of disease activity, no significant correlation was observed between the levels of serum IL-35 and SLEDAI-2K scores (r=0.159, *p*=0.271) (**Figure 2B**).

### IL-12Rβ2^+^ and gp130^+^ Cells in Lymphocyte Subpopulations of Healthy Controls and SLE Patients

The proportion of IL-12Rβ2^+^ cells did not differ significantly between healthy controls and SLE patients in all three lymphocyte subpopulations investigated *i.e.* CD3^─^CD4^─^ (*p*=0.483), CD3^+^CD4^─^ (*p*=0.126) and CD3^+^CD4^+^ (*p*=0.236) (**Supplementary Figures 1A–C**). Likewise, the proportion of gp130^+^ cells did not differ significantly between the healthy controls and SLE patients in the lymphocyte subpopulations (**Supplementary Figures 1A–C**) *i.e.* CD3^─^CD4^─^ (*p*=0.701), CD3^+^CD4^─^ (*p*=0.080) and CD3^+^CD4^+^ (*p*=0.196) (**Supplementary Figures 1A–C**). The proportion of CD3^+^CD4^+^gp130^+^IL-12Rβ2^+^ or CD3^+^CD4^+^gp130^+^IL-12Rβ2^─^ cells also did not differ significantly between healthy controls and SLE patients, *p*=0.230 and *p*=0.396, respectively (**Supplementary Figure 1D**).

**Figure 3:**
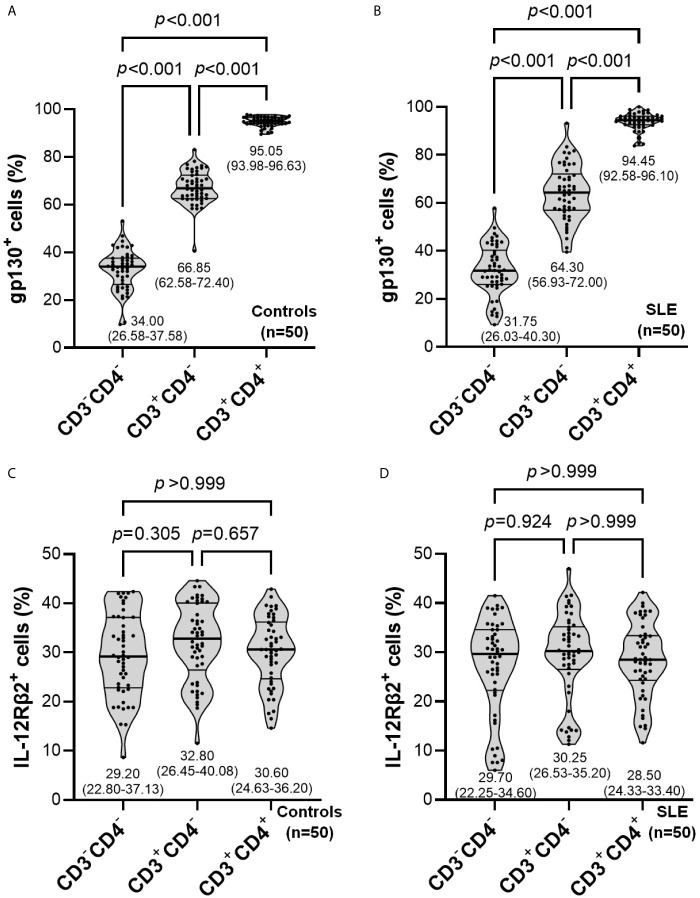
Comparisons of the proportion of gp130^+^ **(A, B)** or IL-12RB2^+^ **(C, D)** cells in lymphocyte subpopulations within controls (n=50) or SLE patients (n=50). The inset numbers below each violin plot represent median (IQR).

The proportion of gp130^+^ cells was significantly higher in CD3^+^CD4^+^ compared with CD3^+^CD4^─^ and CD3^─^CD4^─^ populations in both healthy controls (**Figure 3A**) and SLE patients (**Figure 3B**) (*p*<0.001 for all comparisons). No significant difference was observed for the proportion of IL-12Rβ2^+^ cells in these lymphocyte subpopulations between healthy controls (**Figure 3C**) and SLE patients (**Figure 3D**).

Next, we compared our observations of high gp130 frequency on CD3^+^CD4^+^ T cells with recent transcriptomics profiling data of immune cells by RNA-seq publicly-available on Human Protein Atlas (HPA) database (http://www.proteinatlas.org). HPA’s Consensus Dataset, a combination of three transcriptomics datasets *i.e.* HPA, Genotype-Tissue Expression (GTEx) and Functional Annotation of the Mammalian Genome (FANTOM5), demonstrated that *IL6ST* expression was the highest in naïve CD4^+^ T cells followed by naïve CD8^+^ T cells, memory CD4^+^ T cells and other immune cell subsets (**Supplementary Figure 2A**). In addition, two recent independent RNA-seq datasets of immune cells by Schmiedel et al. (41) and Monaco et al. (42) also demonstrated that *IL6ST* expression was the highest in naïve CD4^+^ T cells followed by multiple other T cell subsets, and *IL6ST* was lowly expressed in other immune cell subsets such as monocytes, B cells and NK cells (**Supplementary Figures 2B, C**).

### Association of IL-12Rβ2^+^ and gp130^+^ Cells With SLEDAI-2K Scores

**Figure 4:**
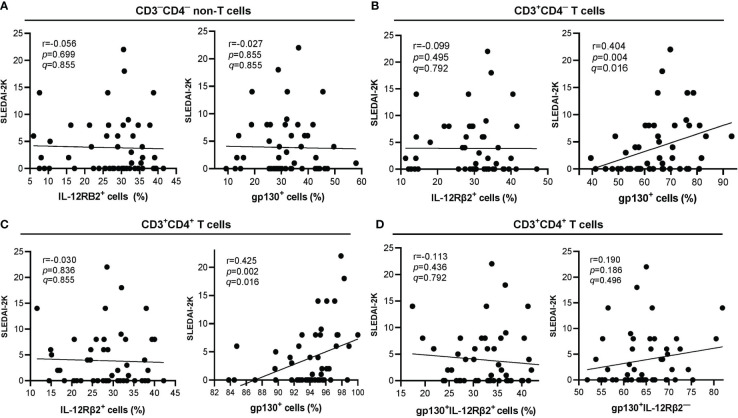
Correlation of lymphocyte subpopulations expressing surface IL-12Rβ2 or gp130 (in percentage) with SLEDAI-2K scores in SLE patients (n=50). **(A)** Correlation of CD3^─^CD4^─^ non-T cells expressing surface IL-12Rβ2^+^ (left) or gp130^+^ (right) with SLEDAI-2K scores. **(B)** Correlation of CD3^+^CD4^─^ T cells expressing surface IL-12Rβ2^+^ (left) or gp130^+^ (right) with SLEDAI-2K scores. **(C)** Correlation of CD3^+^CD4^+^ T cells expressing surface IL-12Rβ2^+^ (left) or gp130^+^ (right) with SLEDAI-2K scores. **(D)** Correlation of CD3^+^CD4^+^ T cells expressing surface gp130^+^IL-12Rβ2^+^ (left) or gp130^+^IL-12Rβ2^─^ (right).

gp130^+^ cells were positively correlated with SLEDAI-2K scores in CD3^+^CD4^+^ (r=0.425, *p*=0.002) and CD3^+^CD4^─^ (r=0.404, *p*=0.004) populations but not in CD3^─^CD4^─^ (r=0.027, *p*=0.855) population (**Figures 4A–C**). Both of these associations remained significant after BH correction, and their *q*-values were <0.05 *i.e.* gp130^+^ cells with SLEDAI-2K scores on CD3^+^CD4^─^ T cells (*q*=0.016) (**Figure 4B**) or CD3^+^CD4^+^ T cells (*q*=0.016) (**Figure 4C**). No correlation was observed between IL-12Rβ2^+^ cells with SLEDAI-2K scores in all these three populations (**Figures 4A–C**). The proportion of CD3^+^CD4^+^gp130^+^IL-12Rβ2^+^ or CD3^+^CD4^+^gp130^+^IL-12Rβ2^─^ cells was not significantly correlated with SLEDAI-2K scores (**Figure 4D**).

It was previously reported that plasma IL-35 and soluble gp130 levels were positively correlated with each other in SLE patients (17), and as such, we next examined the correlation of serum IL-35 levels with CD3^+^CD4^+^gp130^+^ or CD3^+^CD4^─^gp130^+^ populations (*i.e.* populations with SLEDAI-2K relevance) in our cohort of SLE patients. Serum IL-35 levels were not associated with CD3^+^CD4^+^gp130^+^ (r=-0.069, *p*=0.635) or CD3^+^CD4^─^gp130^+^ (r=-0.057, *p*=0.696) populations. Additionally, serum IL-35 levels were also not associated with CD3^+^CD4^+^IL-12Rβ2^+^ (r=-0.119, *p*=0.410) or CD3^+^CD4^─^IL-12Rβ2^+^ (r=-0.223, *p*=0.120) populations (**Supplementary Figure 3**).

### *IL6ST* Expression Is Associated With Treg, T_CM_, and CCR7^+^ T Cells Gene Sets

**Figure 5:**
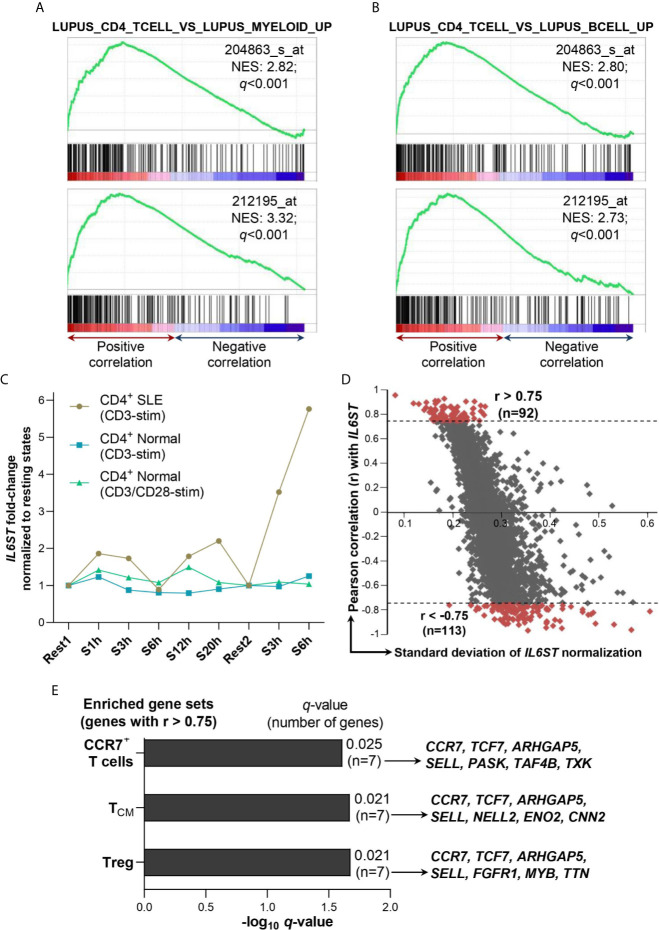
Association of *IL6ST* expression with T cell subsets in SLE patients. **(A)** *IL6ST* expression levels were significantly associated with SLE CD4^+^ T cells compared with SLE myeloid cells gene set (GSE10325_LUPUS_CD4_TCELL_VS_LUPUS_MYELOID_UP; ID: M3087) according to GSEA utilizing two independent *IL6ST* probes (204863_s_at and 212195_at) in SLE patients (n=8; GSE4588 dataset). **(B)** *IL6ST* expression levels were significantly associated with SLE CD4^+^ T cells compared with SLE B cells gene set (GSE10325_LUPUS_CD4_TCELL_VS_LUPUS_BCELL_UP; ID: M3084) according to GSEA utilizing two independent *IL6ST* probes (204863_s_at and 212195_at) in SLE patients (n=8; GSE4588 dataset). **(C)** *IL6ST* expression in CD4^+^ T cells of SLE patients or normal controls (n=9 for each group) stimulated with anti-CD3 or anti-CD3/anti-CD28 (control samples only) in GSE1057 dataset. *IL6ST* expression in each stimulated samples was normalized to their corresponding resting states *i.e.* S1h (stimulated for 1 hour), S3h, S6h, S12h and S20h were normalized to Rest1, while the second group of S3h and S6h (each stimulated after 3 days of resting) were normalized to Rest2 (unstimulated for 3 days). CD4^+^ SLE: CD4^+^ T cells derived from SLE patients; CD4^+^ Normal: CD4^+^ T cells derived from normal subjects; CD3-stim: Stimulated with anti-CD3 antibody; CD3/CD28-stim: Stimulated with anti-CD3 and anti-CD28 antibodies. **(D)** Correlation of *IL6ST* with 9,128 genes in SLE patients (n=9) *i.e.* Rest1, S1h, S3h, S6h, S12h and S20h, and Rest2, S3h and S6h as described previously. The Pearson r value ±0.75 was used as the cut-off to define positive (>0.75) or negative (<-0.75) association with *IL6ST*. **(E)** Gene sets enriched from genes positively associated with *IL6ST* expression (r>0.75) as shown in **(D)** according to ImmuneMap database. All *q*-values were obtained with the Benjamini-Hochberg correction. NES, Normalized enrichment score.

Next, we examined the SLE immune gene sets (*i.e.* immune gene sets in SLE samples derived from the C7 immunologic signature gene sets of MSigDB collections) associated with gp130 expression at the transcript levels (*i.e. IL6ST* gene) through GSEA of a GEP dataset of CD4^+^ T cells isolated from SLE patients (n=8; GSE4588). *IL6ST* expression levels were positively associated with genes upregulated in CD4^+^ T cells vs myeloid cells of SLE patients according to two independent *IL6ST* probes, 204863_s_at (*q*<0.001) and 212195_at (*q*<0.001) (**Figure 5A**). The positive association with *IL6ST* expression profile also occurred with genes upregulated in CD4^+^ T cells vs B cells of SLE patients (*q*<0.001 for both *IL6ST* probes) (**Figure 5B**).

In an independent GEP study of CD4^+^ T cells of SLE patients (n=9) in resting states vs stimulation with anti-CD3, *IL6ST* had been shortlisted as one of 591 genes that were differentially expressed in these SLE T cells (33). Hence, we retrieved the GEP dataset for re-analysis where *IL6ST* expression (probe no. 3750) was indeed augmented upon stimulation at all timepoints investigated (*i.e.* 1h, 3h, 12h and 20h except 6h post-stimulation) compared with the first resting state (Rest1) (**Figure 5C**). *IL6ST* expression was induced more remarkably when the CD4^+^ T cells from SLE patients were stimulated again with anti-CD3 after the second rest for 3 days (Rest2) (**Figure 5C**). We subsequently calculated the Pearson correlation coefficient of all genes present in the GEP dataset with *IL6ST* expression values across all resting and stimulated states of the SLE CD4^+^ T cells samples (n=9). This enabled us to shortlist for genes with positive (r>0.75; n=92 genes) or negative (r<-0.75; n=113 genes) association with *IL6ST* expression (**Figure 5D** and **Supplementary Table 2**). Functional enrichment analysis for SLE immune gene sets of the positively-associated *IL6ST* genes yielded three significantly enriched gene sets *i.e.* Treg cells (*q*=0.021), T_CM_ (*q*=0.021) and CCR7^+^ T cells (*q*=0.025) gene sets (**Figure 5E**). In particular, four core genes contributed to the enrichment of all three gene sets *i.e. CCR7*, *TCF7*, *ARHGAP5* and *SELL* (**Figure 5E**). No SLE immune gene sets were enriched for genes negatively-associated with *IL6ST* expression.

## Discussion

In this study, we showed that serum IL-35 levels were significantly higher in SLE patients than healthy controls. This finding is in agreement with previous studies which showed elevated levels of IL-35 in active SLE patients (17, 18, 43). However, no correlation was observed between serum IL-35 levels with SLEDAI-2K scores. Moreover, multiple studies have demonstrated that serum IL-35 levels were decreased in SLE patients compared with healthy controls (44–46). The observed differences between these studies, and the lack of IL-35 correlation with SLEDAI-2K scores, may be due to the use of glucocorticoids that have been reported to affect IL-35 levels (44) or different profiles of the disease entity (*e.g.* different SLEDAI-2K profiles, anti-dsDNA status, dosage of concomitant steroids or immunomodulatory drugs) in independent cohorts of SLE patients. These potential explanations remain to be validated.

Next, we demonstrated that surface levels of the receptors for IL-35 *i.e.* IL-12Rβ2 and gp130 did not differ significantly between SLE patients and healthy controls in all lymphocyte subpopulations investigated. Interestingly, increasing proportion of CD3^+^CD4^─^ or CD3^+^CD4^+^ T cell populations was significantly associated with higher SLEDAI-2K scores. This is comparable with past study demonstrating that untreated SLE patients had higher surface levels of gp130 on CD4^+^ T cells than inactive or stable SLE patients (47). Subsets of CD3^+^CD4^+^ cells have been shown to be expanded in active SLE (48, 49), and overactive or chronically active T cells are crucial in the pathogenesis of SLE (50, 51). In addition, correlation with SLEDAI-2K scores was not observed in our cohort of patients when surface levels of IL-12Rβ2 were taken into account *i.e.* gp130^+^IL-12Rβ2^+^ or gp130^+^IL-12Rβ2^─^ in CD3^+^CD4^+^ T cells. This indicates that association of surface gp130 levels with higher disease activity in SLE patients was independent of IL-12Rβ2. Furthermore, gp130 subunit is shared with other family members of IL-6 and IL-12 cytokines that bind to and activate gp130, leading to enhanced inflammation in SLE (52, 53).

Our observations of high gp130 frequency on CD3^+^CD4^+^ T cells are consistent with recent transcriptomics profiling data of immune cells by RNA-seq publicly-available on HPA database (54–56). We further showed that *IL6ST* expression in CD4^+^ T cells of SLE patients was increased upon anti-CD3 stimulation in the GSE1057 GEP dataset. This is comparable with our observations of increased SLEDAI-2K scores with augmented gp130^+^ proportion in CD3^+^CD4^+^ T cells. Moreover, Treg, T_CM_ and CCR7^+^ cells gene sets were significantly enriched by genes positively-associated with *IL6ST* expression upon anti-CD3 stimulation, suggesting that these CD4^+^ T cell subpopulations play roles in the exacerbation of SLE disease activities. Indeed, gp130 expression was recently shown to be increased in a specific Treg subset termed as FOXP3-positive, suppression-negative (FPSN) subpopulation where conventional Tregs suppressive function was diminished (29). Human Treg cells that expressed gp130 displayed reduced suppressive function *ex vivo*, indicating that gp130 transmitted inflammatory signals that inhibited the suppressive capability of Treg cells, and subsequent blockade of gp130 was able to restore the suppressive capacity of Treg cells to normal levels (29). Such Treg subpopulation with diminished suppressive activities had previously been identified (57, 58).

Regarding T_CM_ subset, it has been demonstrated that CD4^+^ T cells expressing gp130 modulates inflammatory immune response by promoting early development of pathogenic Th17 cells and inflammation (59). In line with this observation, CD4^+^ stem cell-like memory T (T_SCM_) cells were found to be higher in SLE patients compared with controls, and stimulated T_SCM_ cells from SLE patients could differentiate into follicular helper T cells that subsequently increased autoantibodies production by B cells (60). Furthermore, CCR7 is expressed in both Treg and T_CM_ subpopulations (61–64), and this at least partially explains the enriched CCR7^+^ cells gene sets by genes positively-associated with *IL6ST* expression. Collectively, gp130 may be involved in the activation of both CD4^+^ Treg FPSN and T_CM_ subpopulations that aggravate SLE disease course, and this represents fertile areas for future investigations. We acknowledge the limitations of our study as follows: 1) Only four markers (*i.e.* CD3, CD4, gp130 and IL-12Rβ2) to define the T cell subsets without including markers to identify Tregs and Bregs that produce IL-35, as well as their subpopulations particularly Tregs without functional suppressive capacity typically characterized by FOXP3^+^Helios^─^ expression (29, 58, 65); 2) We did not include a viability dye to distinguish dead cells; 3) Our cohort of healthy controls were not completely matched for age and gender with SLE patients. However, all were adult subjects in both groups (within 18-59 years old), and at least 82% of the subjects in both groups were females.

In recent years, progress has been made to identify and characterize gp130 inhibitors to attenuate its signaling in autoimmune diseases and malignancies. SC144 is an orally active, small molecule gp130 inhibitor with high potency against human ovarian cancers without toxicity to normal tissues, and this was achieved by binding to gp130 that abrogated subsequent STAT3 activation (66). Bazedoxifene, approved for use as a selective estrogen receptor modulator for treatment of osteoporosis, was recently shown to be a novel inhibitor of IL-6/gp130 protein-protein interactions. This led to inhibited STAT3 phosphorylation and suppressed growth of pancreatic cancer cells with intact IL-6/gp130 signaling in both *in vitro* and *in vivo* settings (67). Bazedoxifene also inhibited rhabdomyosarcoma tumor growth *in vivo via* blocking of gp130 signaling and subsequent attenuation of STAT3 phosphorylation and transcription of STAT3 downstream genes (68). Another small molecule inhibitor LMT-28 (a derivative of oxazolidinone) was recently demonstrated to suppress IL-6 signaling by directly binding gp130, resulting in alleviation of inflammatory diseases such as RA and inflammatory bowel disease (69). LMT-28 could also suppress the differentiation of pro-inflammatory Th17 cells in human PBMCs by blocking gp130 and its subsequent STAT3 signaling cascade, and it was proposed as a gp130-specific inhibitor for treatment of RA (70). The availability of gp130-specific inhibitors represents promising avenues for future investigations to therapeutically target gp130 in SLE patients.

Activation of gp130 by its cognate cytokine triggers the JAK/STAT signaling cascade. In recent years, JAK inhibitors have emerged as promising therapeutic agent for SLE in both experimental settings (71, 72) and clinical trials. In particular, baricitinib, a JAK1/2 inhibitor approved for the treatment of RA (73), reduced joint manifestation in non-renal adult SLE patients (n=314) in a phase II randomized controlled trial (RCT) (74). Two multi-center phase III RCTs (NCT03616912 and NCT03616964) are currently assessing the efficacy of baricitinib in adult SLE patients. As multiple cytokines share gp130 subunit as the signal transducer, inhibition of JAK/STAT pathway allows simultaneous disruption of multiple cytokines’ signaling (75) and JAK inhibitors thus represent potential therapeutic agents to derail gp130 downstream signaling cascades in SLE.

In summary, our study demonstrated significantly higher levels of serum IL-35 in SLE patients. Essentially, CD3^+^CD4^+^ T cells expressing gp130 were most significantly correlated with higher SLEDAI-2K scores and they may be associated with pathogenic Treg or T_CM_ subpopulations in SLE. Our findings suggest that gp130 contributes to increased disease severity in SLE patients and it represents a potential therapeutic target for inhibition in the disease.

## Data Availability Statement

The original contributions presented in the study are included in the article/**Supplementary Material**. Further inquiries can be directed to the corresponding authors.

## Ethics Statement

The studies involving human participants were reviewed and approved by Human Research Ethics Committee of Universiti Sains Malaysia (Ethics approval code no.: USM/JEPeM/17120680). The patients/participants provided their written informed consent to participate in this study.

## Author Contributions

CMCH and KKW conceived the study and recruited research grants. NDMS, AFI, WSWG and CMCH recruited the samples and retrieved the subjects’ clinico-demographical data. NDMS and AFI conducted the experiments. KKW performed and interpreted the bioinformatics analysis. NDMS and KKW designed the study, performed data analysis, generated figures and tables, conducted literature searches, wrote and revised the manuscript. All authors contributed to the article and approved the submitted version.

## Funding

This research project was supported by the Research University grant (1001/PPSP/8012246) by Universiti Sains Malaysia awarded to CMCH and the Research University grant (1001/PPSP/8012349) awarded to KKW.

## Conflict of Interest

The authors declare that the research was conducted in the absence of any commercial or financial relationships that could be construed as a potential conflict of interest.

## Supplementary Material

Supplementary Figure 1 Comparison of lymphocyte subpopulations expressing surface IL-12Rβ2 or gp130 (in percentage) in controls (n=50) vs SLE patients (n=50). ** (A) ** The proportion of surface IL-12Rβ2^+^ (left) or gp130^+^ (right) on CD3^─^CD4^─^ non-T cells. ** (B) ** The proportion of surface IL-12Rβ2^+^ (left) or gp130^+^ (right) on CD3^+^CD4^─^ T cells. ** (C) ** The proportion of surface IL-12Rβ2^+^ (left) or gp130^+^ (right) on CD3^+^CD4^+^ T cells. ** (D) ** The proportion of surface gp130^+^IL-12Rβ2^+^ (left) or gp130^+^IL-12Rβ2^─^ (right) on CD3^+^CD4^+^ T cells. The three lines within each violin plot represent first quartile (bottom line), median (center line) and third quartile (top line). The superscript a or b label adjacent to each *p*-value denotes Mann-Whitney U test or unpaired t-test, respectively.

Supplementary Figure 2
*IL6ST* expression levels in publicly-available RNA-seq datasets as annotated by Human Protein Atlas (HPA). **(A)** *IL6ST* expression levels by RNA-seq according to HPA’s Consensus Dataset that represents combination of three transcriptomics datasets (HPA, GTEx and FANTOM5). **(B, C)** *IL6ST* expression levels by RNA-seq according to Schmiedel et al., 2018 **(B)** and Monaco et al., 2019 **(C)** datasets as annotated by HPA. NX: Normalized eXpression; TPM: Transcripts per million; pTPM: TPM values per sample scaled to a sum of 1 million TPM. Image obtained from Human Protein Atlas (available from v20.proteinatlas.org).

Supplementary Figure 3 Correlation of serum IL-35 concentration (pg/ml) with the proportion of CD3^+^CD4^+^gp130^+^ **(A)**, CD3^+^CD4^─^gp130^+^ **(B)**, CD3^+^CD4^+^IL-12Rβ2^+^ **(C)**, or CD3^+^CD4^─^IL-12Rβ2^+^ **(D)** populations.
