# Supplementary figures and images for: CD3+CD4+gp130+ T Cells Are Associated With Worse Disease Activity in Systemic Lupus Erythematosus Patients
Source: Front Immunol. 2021 Jun 4;12:675250. doi: 10.3389/fimmu.2021.675250 (PMC8213373; doi:10.3389/fimmu.2021.675250)

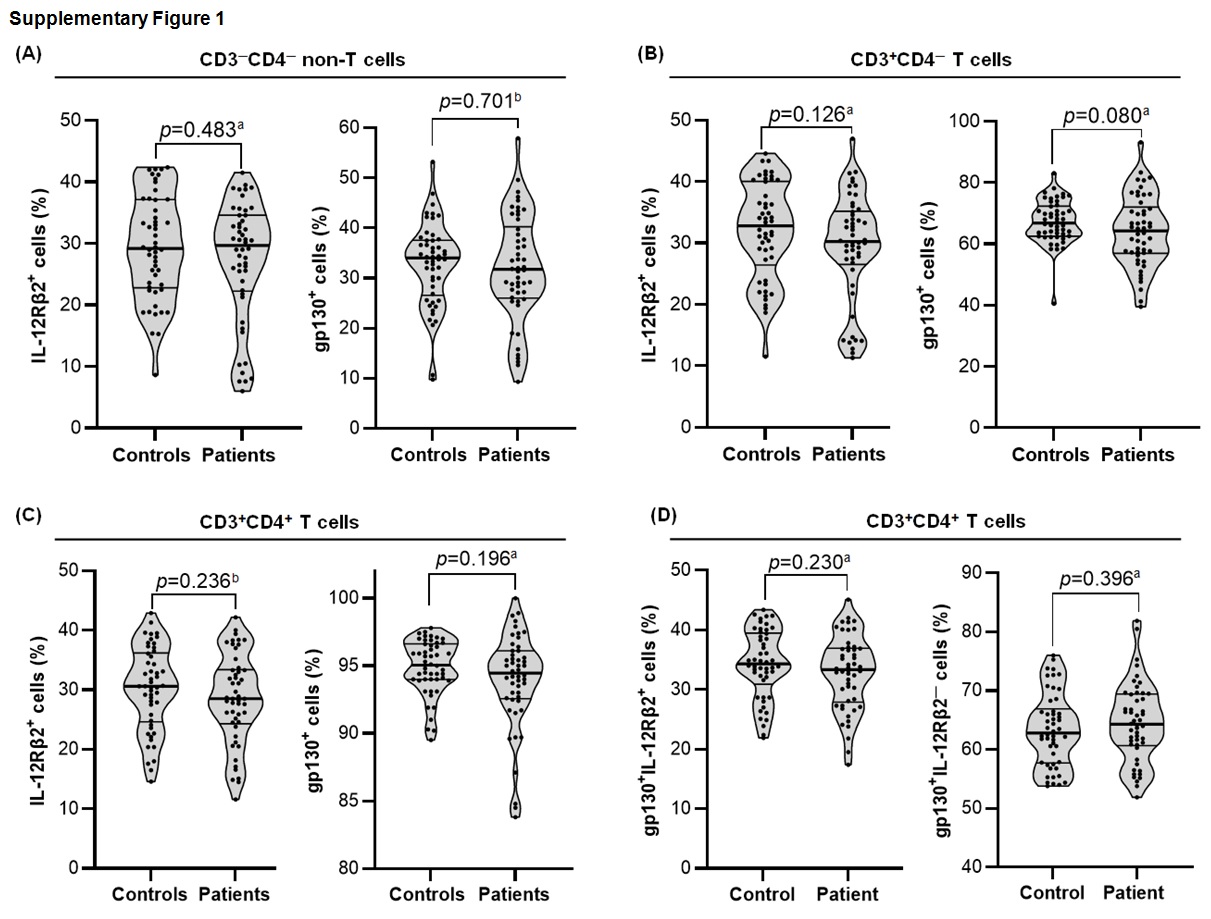

Supplement: Supplementary Figure 1 — Comparison of lymphocyte subpopulations expressing surface IL-12Rβ2 or gp130 (in percentage) in controls (n=50) vs SLE patients (n=50). (A) The proportion of surface IL-12Rβ2+ (left) or gp130+ (right) on CD3─CD4─ non-T cells. (B) The proportion of surface IL-12Rβ2+ (left) or gp130+ (right) on CD3+CD4─ T cells. (C) The proportion of surface IL-12Rβ2+ (left) or gp130+ (right) on CD3+CD4+ T cells. (D) The proportion of surface gp130+IL-12Rβ2+ (left) or gp130+IL-12Rβ2─ (right) on CD3+CD4+ T cells. The three lines within each violin plot represent first quartile (bottom line), median (center line) and third quartile (top line). The superscript a or b label adjacent to each p-value denotes Mann-Whitney U test or unpaired t-test, respectively. [file Image_1.jpeg]

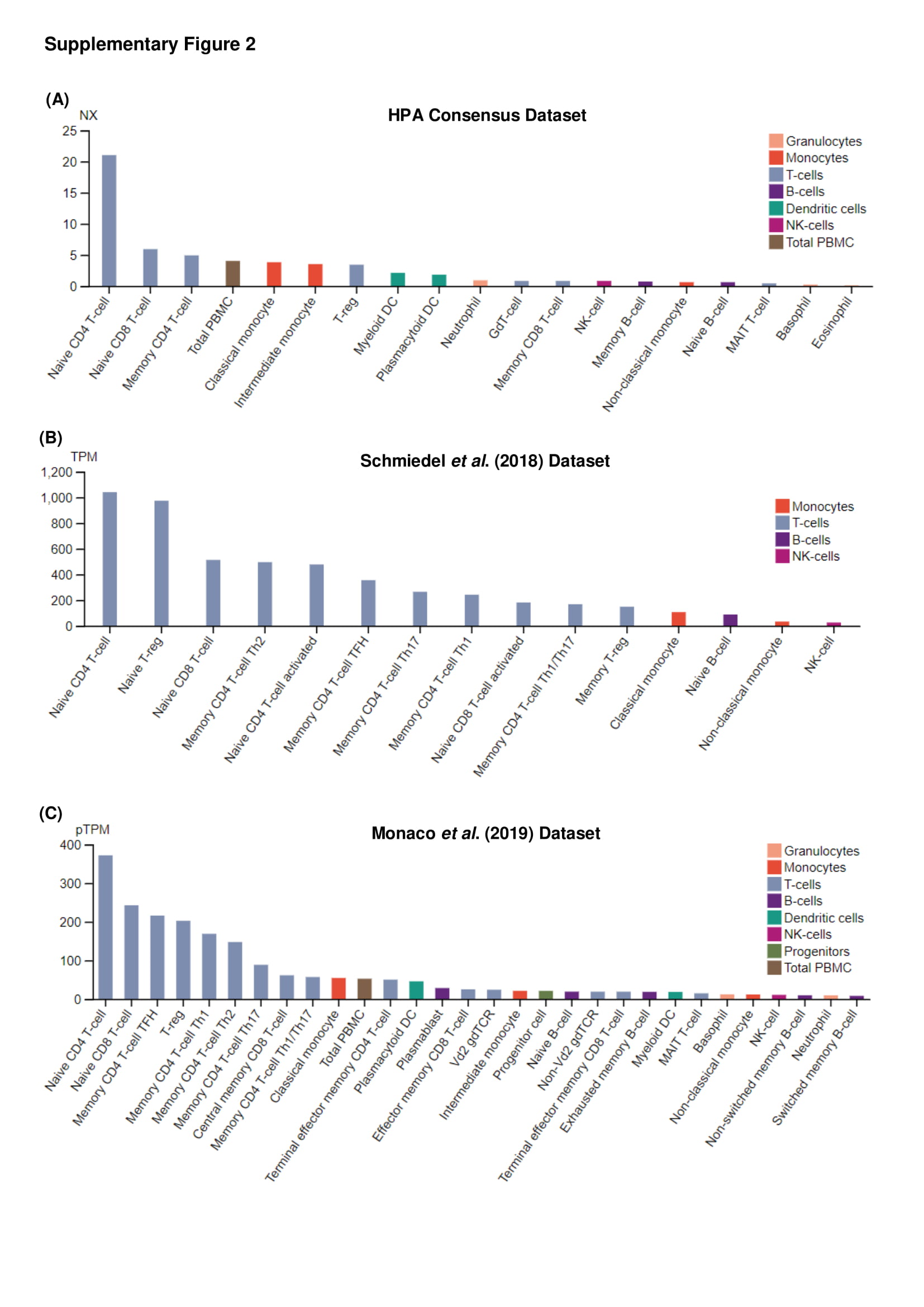

Supplement: Supplementary Figure 2 — IL6ST expression levels in publicly-available RNA-seq datasets as annotated by Human Protein Atlas (HPA). (A) IL6ST expression levels by RNA-seq according to HPA’s Consensus Dataset that represents combination of three transcriptomics datasets (HPA, GTEx and FANTOM5). (B, C) IL6ST expression levels by RNA-seq according to Schmiedel et al., 2018 (B) and Monaco et al., 2019 (C) datasets as annotated by HPA. NX: Normalized eXpression; TPM: Transcripts per million; pTPM: TPM values per sample scaled to a sum of 1 million TPM. Image obtained from Human Protein Atlas (available from v20.proteinatlas.org). [file Image_2.jpeg]

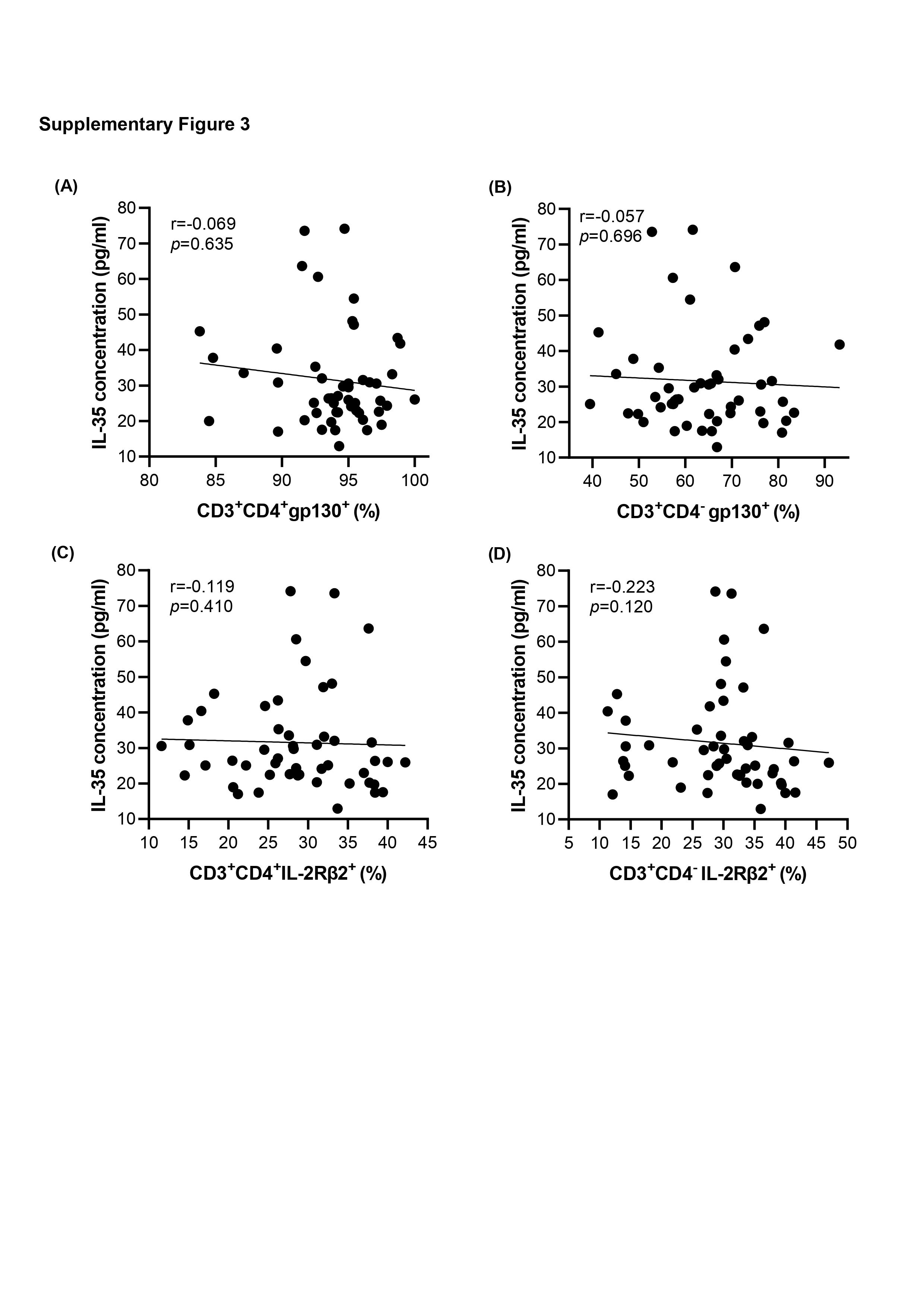

Supplement: Supplementary Figure 3 — Correlation of serum IL-35 concentration (pg/ml) with the proportion of CD3+CD4+gp130+ (A), CD3+CD4─gp130+ (B), CD3+CD4+IL-12Rβ2+ (C), or CD3+CD4─IL-12Rβ2+ (D) populations. [file Image_3.jpeg]
