# Supplementary material for: CD3+CD4+gp130+ T Cells Are Associated With Worse Disease Activity in Systemic Lupus Erythematosus Patients
Source: Front Immunol. 2021 Jun 4;12:675250. doi: 10.3389/fimmu.2021.675250 (PMC8213373; doi:10.3389/fimmu.2021.675250)
Supplement: Supplementary file 4 [file Table_1.docx]

**Supplementary Table 1.** Clinico-demographic characteristics of SLE patients and healthy controls (n=50 in each group).

| **Characteristics** | **Patients, n (%)** | **Controls, n (%)** | ***p*-value** |
| --- | --- | --- | --- |
| **Age (years)**  Median age (IQR) | 34 (26-39.8) | 26.5 (24-31.8) | 0.002 |
| **Gender**  Female  Male | 49 (98.0)  1 (2.0) | 41 (82.0)  9 (18.0) | 0.008 |
| **SLEDAI-2K score**  Median (range)    In remission (SLEDAI: 0)  Intermediate (SLEDAI: 1-3)  Active disease (SLEDAI: ≥4) | 1.6  (range 0-22)  23 (46.0)  7 (14.0)  20 (40.0) | - | - |
| **Serological features**  Antinuclear antibody (ANA)  positive  Anti-dsDNA antibody positive  (>60 IU/ml)  C-reactive protein (CRP)  inflammation state (≥10 mg/l) | 24 (48.0)  34 (68.0)  6 (12.0) | - | - |
| **Medications***  Prednisolone + Hydroxychloroquine + Azathioprine  Prednisolone + Hydroxychloroquine  Prednisolone + Azathioprine  Hydroxychloroquine + Azathioprine  Hydroxychloroquine  Hydrocortisone cream (1%) + Methyl salicylate  Ointment + Loratadine | 13 (26.5)  20 (40.8)  3 (6.1)  4 (8.2)  8 (16.3)  1 (2.1) | - | - |
| **Dosage of medications**^†^  Prednisolone 5 mg  Prednisolone 7.5 mg  Prednisolone 10 mg  Prednisolone 15 mg  Prednisolone 20 mg  Prednisolone 30 mg  Hydroxychloroquine 100 mg  Hydroxychloroquine 200 mg  Hydroxychloroquine 300 mg  Hydroxychloroquine 400 mg  Azathioprine 50 mg Azathioprine 75 mg Azathioprine 100 mg Azathioprine 150 mg Loratadine 10 mg | 21  1  8  2 2 2  1 34 1 9 8 2 6 2 1 | - | - |
| **Clinical features**  Lupus nephritis  Skin manifestations  Cardiac involvement  Neurologic manifestations | 5  4  3  2 | - | - |

*Number of patients with treatment data: 49; ^†^Number of patients with azathioprine dosage
data: 18

**Supplementary Table 2.** List of genes positively (r>0.75) or negatively (r<-0.75) associated with *IL6ST* expression in SLE patients (n=9) at resting and anti-CD3-stimulated states derived from GSE1057 GEP dataset.

| **List of genes positively associated with *IL6ST* expression (r>0.75)** | | | | |
| --- | --- | --- | --- | --- |
| **Pearson** | **Gene** | | **Name** | |
| 0.961588 | *LGMN* | | legumain | |
| 0.931372 | *IPW* | | imprinted in Prader-Willi syndrome | |
| 0.915344 | *ST6GALNAC6* | | ST6 (alpha-N-acetyl-neuraminyl-2,3-beta-galactosyl-1,3)- N-acetylgalactosaminide alpha-2,6-sialyltransferase 6 | |
| 0.91413 | *HSD3B1* | | hydroxy-delta-5-steroid dehydrogenase, 3 beta- and steroid  delta-isomerase 1 | |
| 0.909143 | *MMP10* | | matrix metallopeptidase 10 (stromelysin 2) | |
| 0.908326 | *ITGA2* | | integrin, alpha 2 (CD49B, alpha 2 subunit of VLA-2 receptor) | |
| 0.900889 | *RXRG* | | retinoid X receptor, gamma | |
| 0.893905 | *MT1JP/MT1F* | | metallothionein 1J (pseudogene)/metallothionein 1F | |
| 0.891973 | *ARMCX2* | | armadillo repeat containing, X-linked 2 | |
| 0.887803 | *CCR7* | | chemokine (C-C motif) receptor 7 | |
| 0.870377 | *BCL6* | | B-cell CLL/lymphoma 6 (zinc finger protein 51) | |
| 0.867141 | *KRT16* | | keratin 16 (focal non-epidermolytic palmoplantar keratoderma) | |
| 0.86523 | *GCNT3* | | glucosaminyl (N-acetyl) transferase 3, mucin type | |
| 0.864645 | *TTN* | | titin | |
| 0.864221 | *CHRNA1* | | cholinergic receptor, nicotinic, alpha 1 (muscle) | |
| 0.860349 | *HSPA8* | | heat shock 70kDa protein 8 | |
| 0.859787 | *IFNAR2* | | interferon (alpha, beta and omega) receptor 2 | |
| 0.852334 | *HPCA* | | hippocalcin | |
| 0.849776 | *PSMF1* | | proteasome (prosome, macropain) inhibitor subunit 1 (PI31) | |
| 0.849025 | *IMPDH2* | | IMP (inosine monophosphate) dehydrogenase 2 | |
| 0.846625 | *MYB* | | v-myb myeloblastosis viral oncogene homolog (avian) | |
| 0.845311 | *EDG2* | | endothelial differentiation, lysophosphatidic acid G-protein-coupled  receptor, 2 | |
| 0.840582 | *NELL2* | | NEL-like 2 (chicken) | |
| 0.840042 | *NOL4* | | nucleolar protein 4 | |
| 0.839271 | *ITGAE* | | integrin, alpha E (antigen CD103, human mucosal lymphocyte  antigen 1; alpha polypeptide) | |
| 0.836909 | *PAICS* | | phosphoribosylaminoimidazole carboxylase,  phosphoribosylaminoimidazole succinocarboxamide synthetase | |
| 0.836799 | *POLD3* | | polymerase (DNA-directed), delta 3, accessory subunit | |
| 0.835241 | *AAMP* | | angio-associated, migratory cell protein | |
| 0.8351 | *INADL* | | InaD-like (Drosophila) | |
| 0.832734 | *ABLIM1* | | actin binding LIM protein 1 | |
| 0.827175 | *TCF7* | | transcription factor 7 (T-cell specific, HMG-box) | |
| 0.825866 | *OCLN* | | occludin | |
| 0.820465 | *DHRS3* | | dehydrogenase/reductase (SDR family) member 3 | |
| 0.818625 | *HEPH* | | hephaestin | |
| 0.81549 | *PPIL2* | | peptidylprolyl isomerase (cyclophilin)-like 2 | |
| 0.812906 | *TCEA2* | | transcription elongation factor A (SII), 2 | |
| 0.811495 | *ACTN2* | | actinin, alpha 2 | |
| 0.810869 | *PGR* | | progesterone receptor | |
| 0.80764 | *C18orf1* | | chromosome 18 open reading frame 1 | |
| 0.805764 | *PLAT* | | plasminogen activator, tissue | |
| 0.805362 | *SALL2* | | sal-like 2 (Drosophila) | |
| 0.80474 | *CLCN4* | | chloride channel 4 | |
| 0.80469 | *TXK* | | TXK tyrosine kinase | |
| 0.800348 | *ALDH5A1* | | aldehyde dehydrogenase 5 family, member A1  (succinate-semialdehyde dehydrogenase) | |
| 0.79606 | *MYRIP* | | myosin VIIA and Rab interacting protein | |
| 0.794626 | *STATH* | | statherin | |
| 0.794065 | *SEMA3E* | | sema domain, immunoglobulin domain (Ig), short basic domain,  secreted, (semaphorin) 3E | |
| 0.79328 | *HOMER1* | | homer homolog 1 (Drosophila) | |
| 0.792727 | *SNORA32/ SNORA25/JOSD3* | | small nucleolar RNA, H/ACA box 32/small nucleolar RNA,  H/ACA box 25/Josephin domain containing 3 | |
| 0.791684 | *FGFR1* | | fibroblast growth factor receptor 1 (fms-related tyrosine  kinase 2, Pfeiffer syndrome) | |
| 0.791635 | *MEIS3P1* | | Meis homeobox 3 pseudogene 1 | |
| 0.790133 | *PLCB1* | | phospholipase C, beta 1 (phosphoinositide-specific) | |
| 0.790049 | *ARHGAP5* | | Rho GTPase activating protein 5 | |
| 0.789253 | *HSPA6* | | heat shock 70kDa protein 6 (HSP70B') | |
| 0.789015 | *RPLP0-like* | | ribosomal protein P0-like | |
| 0.788848 | *RPS10* | | ribosomal protein S10 | |
| 0.787581 | *ENO2* | | enolase 2 (gamma, neuronal) | |
| 0.786101 | *C12orf24* | | chromosome 12 open reading frame 24 | |
| 0.78099 | *TMEPAI* | | transmembrane, prostate androgen induced RNA | |
| 0.779719 | *GJB1* | | gap junction protein, beta 1, 32kDa | |
| 0.776006 | *FLJ20323* | | hypothetical protein FLJ20323 | |
| 0.775773 | *SETMAR* | | SET domain and mariner transposase fusion gene | |
| 0.774831 | *DDHD2* | | DDHD domain containing 2 | |
| 0.773317 | *THNSL1* | | threonine synthase-like 1 (S. cerevisiae) | |
| 0.772588 | *MTX3* | | metaxin 3 | |
| 0.771334 | *ITPKB* | | inositol 1,4,5-trisphosphate 3-kinase B | |
| 0.770505 | *PPP2R2C* | | protein phosphatase 2 (formerly 2A), regulatory subunit B,  gamma isoform | |
| 0.769144 | *GRIN3B/CNN2* | | glutamate receptor, ionotropic, N-methyl-D-aspartate  3B/chromosome 19 open reading frame 6/WD repeat  domain 18/calponin 2 | |
| 0.767584 | *PASK* | | PAS domain containing serine/threonine kinase | |
| 0.767317 | *ADM* | | adrenomedullin | |
| 0.76694 | *FVT1* | | follicular lymphoma variant translocation 1 | |
| 0.765862 | *DHX34* | | DEAH (Asp-Glu-Ala-His) box polypeptide 34 | |
| 0.765502 | *TCF7* | | transcription factor 7 (T-cell specific, HMG-box) | |
| 0.764977 | *NFRKB* | | nuclear factor related to kappaB binding protein | |
| 0.763995 | *HOXB2* | | homeobox B2 | |
| 0.762354 | *ATXN7* | | ataxin 7 | |
| 0.760706 | *LOC729998/ LOC654007* | | elongation factor 1 gamma pseudogene/similar to Elongation  factor 1-gamma (EF-1-gamma) (eEF-1B gamma) | |
| 0.759653 | *IGJ* | | immunoglobulin J polypeptide, linker protein for immunoglobulin  alpha and mu polypeptides | |
| 0.755149 | *FGA* | | fibrinogen alpha chain | |
| 0.755037 | *SORBS2* | | sorbin and SH3 domain containing 2 | |
| 0.754999 | *SKIV2L2* | | superkiller viralicidic activity 2-like 2 (S. cerevisiae) | |
| 0.754471 | *LTBP4* | | latent transforming growth factor beta binding protein 4 | |
| 0.753304 | *ITPKB* | | inositol 1,4,5-trisphosphate 3-kinase B | |
| 0.751118 | *TAF4B* | | TAF4b RNA polymerase II, TATA box binding protein  (TBP)-associated factor, 105kDa | |
| 0.750683 | *HSPD1* | | heat shock 60kDa protein 1 (chaperonin) | |
| 0.750256 | *RNF40* | | ring finger protein 40 | |
| **List of genes negatively associated with *IL6ST* expression (r<-0.75)** | | | | |
| **Pearson** | | **Gene** | | **Name** |
| -0.964119 | | *ANKRD36/ KIAA1641* | | ankyrin repeat domain 36/KIAA1641 |
| -0.960931 | | *IL9R* | | interleukin 9 receptor |
| -0.946144 | | *CD63* | | CD63 molecule |
| -0.943089 | | *ITGAX* | | integrin, alpha X (complement component 3 receptor 4 subunit) |
| -0.926852 | | *CDC14A* | | CDC14 cell division cycle 14 homolog A (S. cerevisiae) |
| -0.918905 | | *ACOX1* | | acyl-Coenzyme A oxidase 1, palmitoyl |
| -0.915686 | | *IL32* | | interleukin 32 |
| -0.913008 | | *UTRN* | | utrophin |
| -0.909158 | | *ABHD3* | | abhydrolase domain containing 3 |
| -0.908288 | | *IL10RA* | | interleukin 10 receptor, alpha |
| -0.906064 | | *CLSTN1* | | calsyntenin 1 |
| -0.900034 | | *SLC34A1* | | solute carrier family 34 (sodium phosphate), member 1 |
| -0.899687 | | *NCF4* | | neutrophil cytosolic factor 4, 40kDa |
| -0.895628 | | *LAIR1* | | leukocyte-associated immunoglobulin-like receptor 1 |
| -0.891444 | | *MXD1* | | MAX dimerization protein 1 |
| -0.887584 | | *NFATC3* | | nuclear factor of activated T-cells, cytoplasmic, calcineurin-dependent 3 |
| -0.881078 | | *VPS45* | | vacuolar protein sorting 45 homolog (S. cerevisiae) |
| -0.880562 | | *APLP2* | | amyloid beta (A4) precursor-like protein 2 |
| -0.878629 | | *BCL2* | | B-cell CLL/lymphoma 2 |
| -0.877903 | | *FUCA1* | | fucosidase, alpha-L- 1, tissue |
| -0.877179 | | *ADD3* | | adducin 3 (gamma) |
| -0.876917 | | *STK38* | | serine/threonine kinase 38 |
| -0.874360 | | *GUSB* | | glucuronidase, beta |
| -0.874136 | | *C6orf106* | | chromosome 6 open reading frame 106 |
| -0.872120 | | *NCOA6* | | nuclear receptor coactivator 6 |
| -0.871201 | | *ARPC1B* | | actin related protein 2/3 complex, subunit 1B, 41kDa |
| -0.864944 | | *CENTG1/OS9/ TSPAN31/CDK4* | | centaurin, gamma 1/amplified in osteosarcoma/tetraspanin 31/cyclin-dependent kinase 4 |
| -0.864468 | | *DAZAP2* | | DAZ associated protein 2 |
| -0.858993 | | *TK2* | | thymidine kinase 2, mitochondrial |
| -0.854084 | | *PCNX* | | pecanex homolog (Drosophila) |
| -0.853908 | | *METTL3* | | methyltransferase like 3 |
| -0.850800 | | *EIF4G3* | | eukaryotic translation initiation factor 4 gamma, 3 |
| -0.843084 | | *PDE4A* | | phosphodiesterase 4A, cAMP-specific (phosphodiesterase E2 dunce homolog, Drosophila) |
| -0.840798 | | *BLCAP* | | bladder cancer associated protein |
| -0.837713 | | *IL6* | | interleukin 6 (interferon, beta 2) |
| -0.837265 | | *GATA3* | | GATA binding protein 3 |
| -0.835636 | | *GRLF1* | | glucocorticoid receptor DNA binding factor 1 |
| -0.831894 | | *GATA2* | | GATA binding protein 2 |
| -0.830710 | | *CSF2RB* | | colony stimulating factor 2 receptor, beta, low-affinity (granulocyte-macrophage) |
| -0.822947 | | *IQSEC1* | | IQ motif and Sec7 domain 1 |
| -0.822496 | | *CHD9* | | chromodomain helicase DNA binding protein 9 |
| -0.821194 | | *LOC643319* | | hypothetical LOC643319 |
| -0.819029 | | *KIAA0182* | | KIAA0182 |
| -0.818265 | | *ARG2* | | arginase, type II |
| -0.816320 | | *EGR1* | | early growth response 1 |
| -0.814038 | | *DLG3* | | discs, large homolog 3 (neuroendocrine-dlg, Drosophila) |
| -0.813337 | | *PPP3CA* | | protein phosphatase 3 (formerly 2B), catalytic subunit, alpha isoform |
| -0.813269 | | *FLJ20254* | | hypothetical protein FLJ20254 |
| -0.812424 | | *SLC20A1* | | solute carrier family 20 (phosphate transporter), member 1 |
| -0.811437 | | *MYO1F* | | myosin IF |
| -0.809799 | | *GYG1* | | glycogenin 1 |
| -0.808899 | | *ITGA1* | | integrin, alpha 1 |
| -0.808738 | | *CHD1L* | | chromodomain helicase DNA binding protein 1-like |
| -0.806437 | | *DOK1* | | docking protein 1, 62kDa (downstream of tyrosine kinase 1) |
| -0.805261 | | *TGFBR1* | | transforming growth factor, beta receptor I (activin A receptor type II-like kinase, 53kDa) |
| -0.802254 | | *NOV* | | nephroblastoma overexpressed gene |
| -0.800051 | | *SPCS2* | | signal peptidase complex subunit 2 homolog (S. cerevisiae) |
| -0.796119 | | *QSOX1* | | quiescin Q6 sulfhydryl oxidase 1 |
| -0.795531 | | *BTG2* | | BTG family, member 2 |
| -0.794954 | | *GCS1* | | glucosidase I |
| -0.794591 | | *LAIR1* | | leukocyte-associated immunoglobulin-like receptor 1 |
| -0.792842 | | *PTPN9* | | protein tyrosine phosphatase, non-receptor type 9 |
| -0.792478 | | *ALAS1* | | aminolevulinate, delta-, synthase 1 |
| -0.792051 | | *GNAI2* | | guanine nucleotide binding protein (G protein), alpha inhibiting activity polypeptide 2 |
| -0.791901 | | *CXorf40A* | | chromosome X open reading frame 40A |
| -0.790704 | | *AP1S2* | | adaptor-related protein complex 1, sigma 2 subunit |
| -0.790344 | | *TPM4* | | tropomyosin 4 |
| -0.789835 | | *STK4* | | serine/threonine kinase 4 |
| -0.787933 | | *RPS6KA5* | | ribosomal protein S6 kinase, 90kDa, polypeptide 5 |
| -0.784415 | | *SEPT8* | | septin 8 |
| -0.783634 | | *NEDD8* | | neural precursor cell expressed, developmentally down-regulated 8 |
| -0.783507 | | *PPP3CA* | | protein phosphatase 3 (formerly 2B), catalytic subunit, alpha isoform |
| -0.783288 | | *PIM1* | | pim-1 oncogene |
| -0.783237 | | *CNN2* | | calponin 2 |
| -0.783148 | | *EDF1* | | endothelial differentiation-related factor 1 |
| -0.782775 | | *PTPN18* | | protein tyrosine phosphatase, non-receptor type 18 (brain-derived) |
| -0.781466 | | *LPXN* | | leupaxin |
| -0.779930 | | *FLJ10081* | | hypothetical protein FLJ10081 |
| -0.777528 | | *GSTM2* | | glutathione S-transferase M2 (muscle) |
| -0.777271 | | *TGOLN2* | | trans-golgi network protein 2 |
| -0.777172 | | *RNF103* | | ring finger protein 103 |
| -0.777000 | | *ITM2B* | | integral membrane protein 2B |
| -0.776656 | | *RBM15B* | | RNA binding motif protein 15B |
| -0.776027 | | *HBB* | | hemoglobin, beta |
| -0.774976 | | *LOC442447* | | similar to Chloride intracellular channel protein 4 (Intracellular chloride ion channel protein p64H1) |
| -0.774449 | | *ARAF* | | v-raf murine sarcoma 3611 viral oncogene homolog |
| -0.774083 | | *FAS* | | Fas (TNF receptor superfamily, member 6) |
| -0.773380 | | *TMEM59* | | transmembrane protein 59 |
| -0.772734 | | *C1orf19* | | chromosome 1 open reading frame 19 |
| -0.772723 | | *ITGB5* | | integrin, beta 5 |
| -0.772528 | | *NCSTN* | | nicastrin |
| -0.772427 | | *HERC1* | | hect (homologous to the E6-AP (UBE3A) carboxyl terminus) domain and RCC1 (CHC1)-like domain (RLD) 1 |
| -0.770378 | | *PHTF2* | | putative homeodomain transcription factor 2 |
| -0.769535 | | *SFRS9* | | splicing factor, arginine/serine-rich 9 |
| -0.765299 | | *SLC25A20* | | solute carrier family 25 (carnitine/acylcarnitine translocase), member 20 |
| -0.765118 | | *SDCCAG1* | | serologically defined colon cancer antigen 1 |
| -0.764858 | | *YKT6* | | YKT6 v-SNARE homolog (S. cerevisiae) |
| -0.762011 | | *GAB2* | | GRB2-associated binding protein 2 |
| -0.760840 | | *NDUFS5* | | NADH dehydrogenase (ubiquinone) Fe-S protein 5, 15kDa (NADH-coenzyme Q reductase) |
| -0.759988 | | *PRKAR2A* | | protein kinase, cAMP-dependent, regulatory, type II, alpha |
| -0.759456 | | *LOC728937/ RPS26* | | similar to 40S ribosomal protein S26/ribosomal protein S26 |
| -0.759359 | | *TFCP2* | | transcription factor CP2 |
| -0.758583 | | *VTI1B* | | vesicle transport through interaction with t-SNAREs homolog 1B (yeast) |
| -0.758579 | | *VAV3* | | vav 3 guanine nucleotide exchange factor |
| -0.758016 | | *DENND1C* | | DENN/MADD domain containing 1C |
| -0.756972 | | *MGC71993/ C17orf49* | | similar to DNA segment, Chr 11, Brigham & Womens Genetics 0434 expressed/chromosome 17 open reading frame 49 |
| -0.755244 | | *SH3BP5* | | SH3-domain binding protein 5 (BTK-associated) |
| -0.753362 | | *CHST10* | | carbohydrate sulfotransferase 10 |
| -0.752572 | | *RHOBTB2* | | Rho-related BTB domain containing 2 |
| -0.752191 | | *CLK2* | | CDC-like kinase 2 |
